# Awareness of cervical cancer and its associated socio-demographic factors among Yemeni immigrant women in Malaysia

**DOI:** 10.1186/s12905-023-02172-y

**Published:** 2023-01-16

**Authors:** Meram Azzani, Eshrak Ba-Alawi, Wahib Mohammed Atroosh, Hematram Yadav

**Affiliations:** 1 Department of Public Health Medicine, Faculty of Medicine, Universiti Teknologi MARA, Sungai Buloh Campus, 47000 Sungai Buloh, Selangor Malaysia; 2 Department of Community Medicine, Faculty of Medicine, MAHSA University, Saujana Putra Campus, 42610 Jenjarum, Selangor Malaysia; 3 Department of Parasitology, Faculty of Medicine, University of Malaya, Kuala Lumpur, Malaysia

**Keywords:** Cervical cancer, Socio-demographic factors, Immigrant women, Malaysia

## Abstract

**Background:**

Studies have revealed that a higher proportion of women affected by cervical cancer are from some minority groups of immigrant women. Hence, this study was conducted to assess Yemeni immigrant women’s awareness of cervical cancer and its associated socio-demographic factors.

**Methods:**

A cross-sectional study was conducted among 370 Yemeni women in Selangor and Kuala Lumpur, Malaysia. Data on the awareness of symptoms/signs, risk factors, and screening programme were collected using Cervical Cancer Awareness Measurement (Cervical CAM) questionnaire.

**Results:**

More than 74% of the study participants were unable to recall any warning symptoms/signs, and 73% were unable to recall any risk factors. The factors associated with the awareness of symptoms and risk factors were age (95% CI 4.22–5.22, *p* = 0.039), marital status (95% CI 4.05–7.87, *p* = 0.021), employment (95% CI 3.89–5.77, *p* = 0.046) and the number of children (95% CI 5.33–6.54, *p* = 0.041).

**Conclusion:**

The findings underline the need for public awareness campaigns to improve public awareness of cancer symptoms and risk factors among underserved communities.

## Introduction

Cervical cancer (CC) is a significant public health problem. Even though it is most treatable and preventable, it is still one of the most common causes of cancer deaths among women in numerous developing countries [1]. About 80% of CC cases and an even greater mortality proportion occur in low‐income countries [2] due to the lack of awareness and the absence of efficient CC screening programs in these settings [3].

In Yemen, the age-standardized incidence rate of cervical cancer cases attributable to HPV per 100,000 was 2.5% in 2020 with a mortality rate of 1.8% [4]. A study done among 283 females attending the gynecological outpatient clinic in Sana’a (The capital of Yemen) found that 30.5% of study participants did not know any risk factors of cervical cancer. However, some of the women knew that HPV (42.3%), multiple sexual partners (36.2%), hereditary (29.6%), and long use of contraceptive pills (29.1%) could be causes of cervical cancer. In addition, 59% and 18% of the participant knew that annual screening and vaccination against HPV infection, respectively, are preventative methods for cervical cancer [5].

In Malaysia, there has been a significant reduction in the incidence, prevalence, and mortality of CC in the last 20 years due to the implementation of a free-of-charge Pap Smear screening program for all Malaysian women in public hospitals and other health facilities [6]. While the consequences of CC have decreased in most countries following the Pap smear availability for the early diagnosis of cancerous changes, CC is still leading to devastating outcomes among various minority groups including immigrants [7]. Some minority groups such as immigrants may not be aware of the available screening programme and may suffer from late-stage CC that can lead to poor health consequences or even death [8]. A study conducted among Iraqi immigrant women staying in Malaysia found that lack of awareness of CC, the availability of Pap smear and the cost of screening services are the reasons for not doing a Pap test [9]. Other studies that have been conducted in high-income countries such as the United States of America (USA) have revealed that a higher proportion of women who suffered from CC are from some minority groups of immigrant women [10]. In Malaysia, the Pap smear test is available for immigrants as a screening tool in public hospitals and private clinics. Hence, healthy women who are willing to undertake regular screening for CC have to pay out of pocket for Pap smear test. Early CC symptoms might not be painful or affect the functioning and may not be predicted as warning signs of CC or elicit help-seeking behavior [11]. Knowledge and awareness of CC are fundamental to improve participation in CC prevention and control. Therefore, it has been found that raising awareness of the warning symptoms and signs of CC and encouraging early presentation could reduce patient-attributable delay in the diagnosis of cancer and reduce mortality [12]. Hence, this study aimed to determine the level of awareness of CC and its socio-demographic variances among Yemeni immigrants in Malaysia.

## Methods

### Study setting

There is high immigration of Yemeni people to Malaysia since the war in Yemen in 2015, where the current total number of Yemeni women in Malaysia aged 18 years and above reached 7742 and they are from almost all the states of Yemen (Source: Yemeni Embassy in Kuala Lumpur). The majority of them (7124, 92%) are between 18 and 50 years old and the rest (618, 8%) are above 50 years. Therefore, a cross‐sectional exploratory survey was carried out in Malaysia among Yemeni women who consented to participate in the study and stay in Selangor and Kuala Lumpur, Malaysia, from 1 April 2019 to 31 June 2019. Data were collected using a face-to-face interview using a semi-structured questionnaire. While the Yemeni registry from Yemen embassy contains data on all Yemeni residents in Malaysia, women of a particular age group (18 and above) were randomly selected to participate in this study. However, the response rate was very low (15%). To account for the non-response rate, the researcher approached the areas with a high Yemeni population and met the Yemeni women in the prayer room of their condominium and asked them to participate conveniently in this study. Adequate information was provided to each woman via a participant information sheet.

### Sample size calculation

The sample size was calculated using the Open Epi program version 3.01. The calculated sample size was 367; this sample size assumed that 50% of immigrant women could recall at least one symptom or risk factor of CC with a 95% confidence interval and an 80% power. We inflated the sample size by considering a 20% non-response rate as a result the total sample size was 441.

### Study tool

The survey was conducted by using a validated modified Arabic version of the CC Awareness Measure (Cervical CAM) [13]. The CAM questionnaire was developed by the Health Behavior Research Centre at University College London (UCL), in collaboration with the UK Department of Health Cancer Team and The Eve Appeal, with funding from The Eve Appeal (Cancer Research UK, 2008). The questionnaire was pretested among 20 Yemeni women to ensure the simplicity and clarity of the study tool.

CC awareness questionnaire (Cervical CAM) questionnaire is a semi-structured questionnaire containing both open-ended (unprompted) and closed (prompted) questions. It contained eight questions to determine women’s awareness of the warning signs/symptoms and risk factors of CC as an outcome variable. Awareness of cancer warning signs and risk factors was determined by using open-ended questions. These questions were asked to assess the respondents’ recall of as many signs/symptoms and risk factors as possible. Moreover, closed-ended questions were used to assess the participant’s ability to recognize 11 signs/symptoms and 11 risk factors. The 11 symptoms listed in the CAM questionnaire are vaginal bleeding between periods, pain during sex, persistent lower back pain, persistent vaginal discharge, vaginal bleeding after menopause, heavy menstrual periods, persistent diarrhea, persistent pelvic pain, vaginal bleeding during or after sex, blood in the stool or urine and unexplained weight loss. The 11 risk factors are infection with human papillomavirus (HPV), smoking, long-term use of contraceptive pills, having a weakened immune system, infection with chlamydia, having a sexual partner who is not circumcised, starting to have sex at a young age, having many sexual partners, having many children, having a sexual partner with many previous partners, and not going for regular Pap smear.

### Description of study variables

Regarding participants’ ability to recall the warning signs and risk factors of CC, the participants were given the chance to freely answer what they knew about them. Score 1 was given to any of the 11 warning signs/symptoms and 11 risk factors that the respondents recalled. Then scores were summed to give a total ‘recall’ score for each woman. For recognizing the 11 warning signs/symptoms, the recognition questions were of three options; “Yes”, “No” or “Don’t know”, while the response options for recognizing the risk factors were “Strongly disagree”, “Disagree”, “Not sure”, “Agree” or “Strongly agree”. For each symptom/sign, a “Yes” response scored “1”, while a “No” or “Don’t know” response scored “0”. For each risk factor, “Agree” or “strongly agree” scored “1”, while “Strongly disagree”, “Disagree” and “Not sure” scored “0”. This scoring method is the same as that utilized in an earlier published study using the CAM questionnaire [14, 15]. The scores for both the recall and the recognition of the warning signs were summed to get a total awareness score for the warning signs, and the same was done for the recall and recognition of risk factors to get a total awareness score for the risk factors [13]. Awareness of the screening program was also explored which includes questions related to age should women start doing the screening test, practicing the Pap smear test, interested to do a Pap smear test, and if they do Pap test every 3 years regularly [13].

The explanatory variables consisted of socio-demographic factors, namely age, marital status, level of education, employment, and monthly income. The income and age of the participants were collected as an open question then they were categorized. The other variables were collected as categorical variables; marital status (single, married and divorced/widowed/separated), level of education (no formal education, primary and secondary, post-secondary, and others (including still studying), and employment (full-time and part-time), unemployed, and others which include retired, still studying, and disabled. Moreover, the spouse’s factors (education level, employment, and monthly income) were categorized as those of the woman. Finally, the cancer history of the woman herself and that of her family and friends were grouped into two categories (no cancer history and has a cancer history).

### Statistical analysis

Data is entered and analyzed using the Statistical Package for Social Sciences 23(SPSS 23). Descriptive statistical analysis was utilized to determine the socio-demographic characteristics of study participants. Means and standard deviation for numerical variables and frequency and percentage for categorical data were reported.

In addition, an analysis of variance (ANOVA) was utilized to assess the socio-demographic variances in the awareness score for the warning signs and risk factors of CC [13]. For statistically significant differences between the groups, multiple comparisons**,** showing which groups differed from each other were done using the Turkey post hoc test which is generally the preferred test for conducting post hoc tests on a one-way ANOVA. A *p*-value of < 0.05 was considered statistically significant.

## Results

A total of 370 responses were obtained out of 441 women approached, representing a response rate of 84%.

### Socio-demographic characteristics

Table 1 shows the socio-demographic characteristics of the study participants and their husbands. The mean age of the study respondents was 32.09 ± 8.44 years. The majority of study participants were married (64.9%), followed by a single (29.5%) and divorced (5.6%). Regarding the number of children, most of the married women had less than three children (63.6%), while the remainder of the married women had three or more children (36.4%). In respect of the highest level of education, 52.4% of the women had a post-secondary level of education, 39.4% had no formal, primary, or secondary school education, and only a small proportion had other qualifications (8.1%). A major proportion of the participants were studying or retired (48.1%), followed by unemployed (35.7%). Only a small proportion of the participants were employed (16.2%). As for the occupational category of the employed participants, the women were categorized into professional and managerial (11.6%), and skilled, semi-skilled and unskilled (7.2%). Regarding the income of the employed women, only a few of them were willing to reveal their income (14.5%), and of those, who disclosed this information, most of them had a low income of less than MYR2000 per month (9.5%), while 5.1% had a middle level of income of MYR2000–4500 per month and none were earning a high income.

**Table 1:** Socio-demographic characteristics of respondents (n = 370)

Socio-demographic characteristic	N	%
Mean age (± SD)	32.09 ± 8.44	
*Age groups*		
18–29	139	37.5
30–39	152	41.1
40–50	79	21.4
*Marital status*		
Single/never married	109	29.5
Married	240	64.9
Divorced/widowed/separated	21	5.6
*Education level*		
No formal, primary and secondary	146	39.5
Post-secondary	194	52.4
Others (still studying etc.)	30	8.1
*Living arrangement*		
Own house	29	7.8
Renting	258	69.7
Others	83	22.4
*Number of children*		
< 3	166	63.6
≥ 3	95	36.4
*Employment status*		
Employed (full-time and part-time)	60	16.2
Unemployed	132	35.7
Others	178	48.1
*Occupational category*		
Professional and managerial	43	11.6
Skilled, semi-skilled, and unskilled	27	7.2
Monthly income (MYR)*		
Low (< 2000)	35	9.5
Middle (2000–4500)	19	5.1
High (> 4500)	0	0.0
*Husband’s highest qualification*		
No formal, primary and secondary	73	19.7
Post-secondary	294	79.5
Others	3	0.8
*Husband’s employment status*		
Employed (full-time and part-time)	169	45.7
Unemployed	42	11.4
Others (retired, studying, etc.)	32	8.6
*Occupational category*		
Professional and managerial	74	20.0
Skilled, semi-skilled, and unskilled	103	27.7
*Husband’s monthly income (MYR)*		
Low (< 2000)	91	24.6
Middle (2000–4500)	47	12.7
High (> 4500)	26	7.0
*Prior history of cancer*		
You	3	0.8
Partner	2	0.5
Family members	122	33.0
Friends	99	26.7

*Malaysian Ringgit

As regards the husbands’ socio-demographic characteristics, in terms of the level of education 79.5% had been awarded a post-secondary level of education, while 19.7% had no formal, primary or secondary school education and only a low proportion had other qualifications (0.8%). As regards the employment of the husband, most were in full-time or part-time employment (45.7%), while 11.4% were unemployed and 8.6% were in the ‘others’ category, which included retired and students. As regards the occupational category, 20% of the husbands were in professional and managerial employment and 27.8% were skilled, semi-skilled and unskilled. The income of the husbands was categorized into low (< MYR2000), middle between MYR2000–4000, and high > MYR4500, and 24.6%, 12.7%, and 7.0% were in these categories, respectively. Regarding the women participants’ history of cancer, 0.8% of the participants themselves had a history of cancer, while they reported a history of cancer in partners (0.5%), family members (33.0%), and friends (26.7%).

### Awareness of signs/symptoms and risk factors of cervical cancer

Concerning the level of awareness of the signs and symptoms by the recall, the most common symptom was vaginal bleeding between periods (19.5%), followed by menstrual periods heavier or longer than usual (17.0%), while the level of awareness exhibited among the participants for persistent vaginal discharge was (7.6%), persistent lower back pain (5.4%) and vaginal bleeding after the menopause (4.3%). The recall awareness for other signs and symptoms was very poor; for example, discomfort or pain during sex (2.2%), persistent diarrhea (0.3%), persistent pelvic pain (2.4%), vaginal bleeding during or after sex (1.9%), blood in the stool or urine (1.4%), and unexplained weight loss (0.5%). On the other hand, the percentage scores for awareness by recognition among the participants were higher: vaginal bleeding between periods (50.0%), vaginal bleeding after menopause (55.1%), persistent vaginal discharge (39.7%), discomfort or pain during sex (31.4%), vaginal bleeding during or after sex (15.9%), persistent pelvic pain (35.9%), blood in the stool or urine (28.4%), unexplained weight loss (30.3%) and persistent diarrhea (14.6%). In general, there was a poor level of awareness by recall as compared to recognition of all the signs and symptoms of CC (Table 2).

**Table 2:** Awareness of warning signs/symptoms and risk factors of cervical cancer

Signs and symptoms	Recall		Recognition	
	n	(%)	n	(%)
Vaginal bleeding between periods	72	(19.5)	185	(50.0)
Persistent lower back pain	20	(5.4)	88	(23.8)
Persistent vaginal discharge	28	(7.6)	147	(39.7)
Discomfort or pain during sex	8	(2.2)	116	(31.4)
Menstrual periods heavier or longer than usual	63	(17.0)	126	(34.1)
Persistent diarrhoea	1	(0.3)	54	(14.6)
Vaginal bleeding after the menopause	16	(4.3)	204	(55.1)
Persistent pelvic pain	9	(2.4)	133	(35.9)
Vaginal bleeding during or after sex	7	(1.9)	59	(15.9)
Blood in the stool or urine	5	(1.4)	105	(28.4)
Unexplained weight loss	2	(0.5)	112	(30.3)
*Risk factors*				
HPV infection	2	(0.5)	164	(44.3)
Smoking	37	(10.0)	212	(57.3)
Weakened immune system	19	(5.1)	245	(66.2)
Use of contraceptives	31	(8.4)	196	(53.0)
Infected with chlamydia	0	(0.0)	150	(40.5)
A sexual partner who is not circumcised	0	(0.0)	94	(25.4)
Starting sex at a young age	6	(1.6)	93	(25.1)
Many sexual partners	5	(1.4)	196	(53.0)
Having many children	8	(2.2)	82	(22.2)
A sexual partner with many previous partners	2	(0.5)	179	(48.4)
Not going for a regular pap smear	23	(6.2)	243	(65.7)

Moreover, the awareness of the risk factors of CC by recall was also very low in comparison to awareness by recognition. For instance, approximately 100% of the women could not recall some risk factor that could lead to having CC, such as a sexual partner who is not circumcised or having been infected with chlamydia. The most recalled risk factors were smoking (10.0%), use of contraceptives (8.4%), and not going for a regular pap smear (6.2%), followed by a weakened immune system (5.1%). Regarding the recognition of risk factors, the weakened immune system was the most recognized factor (66.2%), followed by not going for a regular pap smear (65.7%) and smoking (57.3%). The same proportion of respondents recognized that the use of contraceptives and having many sexual partners were risk factors for CC (53%), followed by the other factors, in descending order of recognition: a sexual partner with many previous partners (48.4%), HPV infection (44.3%), infected with chlamydia (40.5%), a sexual partner who is not circumcised (25.4%), starting sex at a young age (25.1%) and having many children (22.2%) (Table 2).

### Total awareness scores for signs and symptoms and risk factors

Regarding the total awareness score of the participants with respect to their recall and the recognition of the signs and symptoms and the risk factors of CC, it found that the women have poor awareness of CC symptoms and risk factors by recall as compared to recognition (mean = 0.62 vs 3.59 and 0.35 vs 5.01) for the signs and symptoms and the risk factors, respectively.

### Awareness of the cervical cancer screening test

Table 3 shows the awareness of the CC screening test among the study participants. From the table, most of the women stated that they were interested in doing a CC screening test (67.6%). However, regarding the age at which women should start having the test, awareness was poor, in which out of the 370 participants, about 37.0% answered “I don’t know” while 22.2% answered between the age of 18 and 25 years (which is the correct answer). Other participants gave a variety of age points: > 25 years (5.9%), ≥ 30 years (12.2%), and ≥ 40 years (12.4%). Furthermore, other poor answers that were given were at the age of marriage (4.5%), at menopause (2.4%), and when feeling the pain of symptoms that can indicate CC (0.3%).

**Table 3:** Awareness of the cervical cancer screening test

Variable	N	%
*Awareness of cervical cancer screening test*		
Yes	250	67.6
No	37	10.0
I don’t know	83	22.4
*Age of women should start doing the test*		
18–25 years	82	22.2
> 25 years	22	5.9
≥ 30 years	45	12.2
≥ 40 years	46	12.4
At the age of marriage	17	4.5
At menopause	9	2.4
Unrelated to age	11	2.9
When a woman feels pain	1	0.3
I don’t know	137	37.0
*Practiced the pap smear test*		
Yes	36	9.7
No	323	87.3
I don’t know	11	3.0
*Interested in doing a pap smear test*		
Yes	278	75.1
No	75	20.3
I don’t know	17	4.6
*Pap tests should be done regularly every three years*		
Yes	172	46.5
No	92	24.9
I don’t know	106	28.6

The other three questions about awareness of the screening test were designed to elicit what the participants knew about Pap smear test practices and their practices. A majority of the women stated that they did not practice having a pap test (87.3%), while only 9.7% stated “Yes” and 3.0% answered “I don’t know”. However, 75.1% stated that they were interested in doing the test. As regards the participants’ awareness about how often women should do the test more than 46% said “Yes” (women should have the test regularly every 3 years), whereas 24.9% said “No” (that was not the case), and 28.6% responded “I don’t know”.

### Factors associated with awareness of the warning signs/symptoms and risk factors of cervical cancer

Table 4 shows the ANOVA test results for the factors associated with the recall and recognition of the warning signs and symptoms of CC. From the results in the table, it can be observed that age, marital status, and employment were statistically significant associated factors of awareness of warning signs and symptoms. On the other hand, living arrangement**,** occupational category, number of children, women’s monthly income, and husband-related factors were not statistically significant (*p* > 0.05). In respect of age, there was a statistically significant difference among groups (F = 3.278, *p* = 0.039). A Turkey post-hoc test showed that those aged 30–39 years had higher awareness of signs and symptoms as compared to women aged 18–29 years and those in the 40–50 years age group. As regards to marital status, there was a statistically significant difference among groups (F = 3.3895, *p* = 0.021). A Turkey post-hoc test showed that those who were divorced/widowed/separated had higher awareness of signs and symptoms in comparison to married women (5.95 vs. 4.00). There was no statistically significant difference between divorced/separated and single women (*p* = 0.585). As for the effect of employment, there was a statistically significant difference among groups (F = 3.099, *p* = 0.046). A Turkey post-hoc test showed that those who were employed had higher awareness of signs and symptoms than those who were unemployed (4.83 vs. 3.373). There was no statistically significant difference between employed participants and those in the other category (Table 4).

**Table 4:** Analysis of variance showing the factors associated with the recall and recognition of the warning signs/symptoms, and risk factors of cervical cancer (n = 370)

Variable	Warning signs of cervical cancer			Risk factors of cervical cancer		
	Mean	(95% CI)	ANOVA	Mean	(95% CI)	ANOVA
*Age group (years)*						
18–29	3.88	(3.31–4.44)	F = 3.278	5.29	(4.79–5.79)	F = 1.380
30–39	4.71	(4.22–5.22)	*p* = 0.039*	5.19	(4.73–5.65)	*p* = 0.253
40–50	3.85	(3.22–4.47)		5.84	(5.18–6.50)	
*Marital status*						
Single	4.36	(3.78–4.94)	F = 3.895	5.33	(4.77–5.89)	F = 2.722
Married	4.00	(3.61–4.39)	*p* = 0.021*	5.26	(4.90–5.62)	*p* = 0.067
Divorced/widowed	5.95	(4.03–7.87)		6.80	(5.19–8.42)	
*Living arrangement*						
Own house	3.72	(2.59–4.85)	F = 0.549	4.45	(3.50–5.39)	F = 1.887
Renting	4.20	(3.81–4.58)	*p* = 0.578	5.38	(5.01–5.74)	*p* = 0.153
Others	4.43	(3.72–5.15)		5.67	(5.03–6.32)	
*No of children*						
< 3	4.08	(3.63–4.52)	t = 0.369**	5.16	(4.70–5.60)	t = 4.197**
≥ 3	4.31	(3.65–4.97)	*p* = 0.544	5.94	(5.33–6.54)	*p* = 0.041*
*Education*						
No formal/primary and secondary	3.86	(3.37–4.35)	F = 2.87	5.21	(4.74–5.69)	F = 0.376
Post-secondary	4.58	(4.11–5.04)	*p* = 0.058	5.49	(5.07–5.91)	*p* = 0.687
Others (still studying etc.)	3.56	(2.52–4.60)		5.30	(4.18–6.41)	
*Employment*						
Employed (full-time and part-time)	4.83	(3.89–5.77)	F = 3.099	5.65	(4.79–6.50)	F = 0.332
Unemployed	3.71	(3.17–4.24)	*p* = 0.046*	5.34	(4.84–5.84)	*p* = 0.716
Others (retired, studying, etc.)	4.38	(3.93–4.82)		5.29	(4.88–5.72)	
*Occupational category*						
Professional and Managerial	3.86	(2.98–4.73)	t = − 0.505**	5.39	(4.47–6.31)	t = − 0.550**
Skilled/semi-skilled and unskilled	4.22	(3.02–5.42)	*p* = 0.616	5.77	(4.77–6.78)	*p* = 0.584
*Monthly personal income (MYR)*						
< 2000	3.97	(2.80–5.14)	F = 1.169	5.54	(4.43–6.65)	F = 2.088
2000–4500	3.00	(1.73–4.26)	*p* = 0.285	4.37	(3.46–5.28$$)$$	*p* = 0.155
*Husband’s education level*						
No formal, primary, and secondary	3.53	(2.85–4.21)	F = 1.475	5.16	(4.49–5.83)	F = 0.13
Post-secondary	4.25	(3.78–4.73)	*p* = 0.231	5.31	(4.87–5.76)	*p* = 0.875
Others (still studying, etc.)	3.33	(− 8.92–15.58)		4.66	(− 7.07–16.40)	
*Husband’s employment status*						
Employed (full-time and part-time)	4.11	(3.66–4.58)	F = 0.163	5.49	(5.03–5.95)	F = 0.212
Unemployed	4.36	(3.40–5.31)	*p* = 0.850	5.17	(4.32–6.01)	*p* = 0.809
Others (retired, studying, disabled)	3.97	(2.86–5.09)		5.47	(4.51–6.42)	
*Husband’s occupational category*						
Professional and managerial	3.86	(2.98–4.73)	t = − 0.505	5.39	(4.47–6.31)	t = − 0.550
Skilled, semi-skilled and unskilled	4.22	(3.02–5.42)	*p* = 0.616	5.77	(4.77–6.78)	*p* = 0.584
*Husband’s monthly income (MYR)*						
< 2000	4.09	(3.43–4.74)	F = 1.703	5.60	(4.97–6.23)	F = 0.924
2000–4500	4.70	(3.52–5.88)	*p* = 0.185	5.06	(4.18–5.95)	*p* = 0.399
> 4500	3.19	(2.11–4.27)		4.85	(3.76–5.93)	
*History of cancer among family, friends and relatives*						
Yes	4.12	(− 0.83–0.48)	t = − 0.519**	5.37	(− 0.615–0.602)	t =− 0.020**
No	4.29	(− 0.83–0.48)	*p* = 0.604	5.37	(− 0.614–0.602)	*p* = 0.984

*Significant at *p* < 0.05

**t-test was run for two-group variables

In addition, the ANOVA test results for the independent variables of recall and recognition of the risk factors of CC show that all the socio-demographic factors of women and their husbands were not significant (*p* > 0.05), except for one factor, namely, the number of children (*p* < 0.05). In respect of the number of children, there was a statistically significant difference between groups (t = 4.197, *p* = 0.041) in which women who had three or more children had higher awareness of CC risk factors in comparison to those who had less than three children (mean = 5.94 vs. 5.16) (Table 4).

## Discussion

To the best of our knowledge, this is the first study in Malaysia conducted to evaluate Yemeni immigrant women’s awareness of CC symptoms and risk factors and to determine socio-demographic differences in their level of awareness.

Regarding awareness of the symptoms of CC, the most recalled and recognized symptom was vaginal bleeding between periods. Similarly, studies conducted in Yemen and UK found that the commonly recalled symptom is vaginal bleeding between periods [5, 16, 17]. In contrast, a study done in Tanzania found “Persistent vaginal discharge that smells unpleasant” as the most frequently recalled symptoms [18]. In this study, persistent diarrhoea was recalled by only 0.3% of the respondents, which is similar to a finding of a previous study conducted in England [17]. The findings of various studies highlighted that women only think about the symptoms related to the reproductive tract. This calls for action to improve the knowledge of symptoms of CC especially those unrelated to the reproductive tract.

In regard to awareness of the risk factors, the capacity to recall the 11 risk factors was very low among all participants and this is consistent with previous studies [17, 19]. The risk factors of smoking and the use of contraceptives were the most recalled by the current study sample (10% and 8.4%, respectively) and these results are similar to those in the previous study also [19] and in contrast with other studies which found sexual behavior related factors as the most identified risk factors [16, 20]. This could probably be a result of genuine knowledge of this specific risk factor or could be a result of misconception as women have been known to disproportionately relate birth control pills with many side effects such as infertility and cancer [21]. Though, this needs further studies to support.

It was not surprising to find that among the participants in this study awareness by recall was very poor in respect of HPV being a risk factor (0.5%) because similar results have been reported in previous studies[16, 22]. However, a study performed in Egypt revealed that 32.5% think that CC is caused by a viral infection [23].

In terms of awareness by recognition of risk factors, a weakened immune system was recognized as a risk factor by many of the women in this study (66.2%). A similar result was reported by a study done in Libya [19]. However, a study conducted in Yemen found that the most recognized risk factor (69.5%) among Yemeni women is HPV infection [5]. In addition, our study found that 65.7% of the participants answered that not going for a regular pap test is a risk factor, which is similar to the result of previous studies conducted in Yemen and England [5, 16]. In contrast, having many children was the least recognized risk factor among the women in our study (22.2%). Similarly, the study done in Libya found that having many children is the least recognized risk factor for 11% of women [19]. However, recognition of sex as a risk factor was so poor. These results suggest that despite most of the women knowing that a link exists between CC and not going for a Pap test and HPV, the relationship between sex and HPV is not understood by the majority [16, 24].

In addition, most of the women stated that they did not practice having a pap test (87.3%). Similarly, a study conducted among African immigrant women in Malaysia found that the prevalence of Pap smear uptake among the participants in the past 3 years was 27.2% [25]. This may reflect the low awareness and utilization of screening services among immigrant women in Malaysia. Since screening is a crucial part of early cancer diagnosis and later better prognosis, there is a need for efforts to improve the utilization of screening services.

Age, marital status, and employment were statistically significant associated factors for awareness of the warning signs and symptoms of CC, where middle-aged (30–39 years) women had a higher score of awareness. One plausible explanation for this result could be that the incidence of CC is highest in this age group [26]. And there is evidence that younger women (18–29 years) may be more vulnerable to some risk factors such as being infected with chlamydia [27]. The result of our study is consistent with that of a previous study done in England in which age was found to be a significant predictor [16].

Also, in our study, employed women had a higher awareness score as compared to those in the unemployed and other groups. This is consistent with a study done in Libya [19] in which it was reported that the majority of employed women were highly educated a factor that was employed to justify this finding and which is supported by the arguments provided in other previous studies [16, 19].

However, as regards awareness of the risk factors of CC, our study found that only one factor was significant, namely the number of children, where women with three or more children had a higher awareness score as compared to those who had fewer than three children. Despite the number of children being identified as a risk factor for CC, previous studies did not investigate this factor as a predictor of awareness of CC  [16, 19, 28]. Thus, a comparison with previous findings cannot be made.

The strength of this study is that it approaches an important issue in an under-studied population, and it will have great potential to contribute to the improvement of healthcare for minority immigrant women. However, this study has some limitations. First, when the participants were selected randomly, the non-response rate was high which leads the researcher to change the sampling method to convenient sampling. As a result, the sample may not be representative of the whole community. Second, as the information on screening was self-reported, the study may be subjected to information bias.

## Conclusion

Awareness of the signs and symptoms and the risk factors of CC among immigrant Yemeni women in Malaysia remains poor. Based on the evidence obtained, this study revealed that there is variation in the awareness of the signs and symptoms and the risk factors, and this variation is affected by certain socio-demographic factors. The outcomes of this study emphasize that there is a strong and urgent need to increase women’s awareness about CC symptoms and risk factors and the utilization of screening test.

## Recommendations

A health education campaign should be conducted to maximize awareness of CC prevention among the public. Such a campaign may help immigrant women to better understand CC and the significance of screening, and to identify the warning symptoms and risk factors of CC. Furthermore, health education through healthcare providers could improve the response to a preventive health service program for CC. The allocation of a special fund to make CC screening available for immigrant women in public healthcare facilities for a nominal fee is also recommended.

## Data Availability

The datasets used and/or analyzed during the current study are available from the corresponding author upon reasonable request.
